# Incorporation of Chitosan Microspheres into Collagen-Chitosan Scaffolds for the Controlled Release of Nerve Growth Factor

**DOI:** 10.1371/journal.pone.0101300

**Published:** 2014-07-01

**Authors:** Wen Zeng, Mengyao Rong, Xueyu Hu, Wei Xiao, Fengyu Qi, Jinghui Huang, Zhuojing Luo

**Affiliations:** 1 Department of Orthopaedics, Xijing Hospital, The Fourth Military Medical University, Xi’an, China; 2 Department of Neurosurgery, Tangdu Hospital, The Fourth Military Medical University, Xi’an, China; 3 Department of Clinical Immunology, Xijing Hospital, The Fourth Military Medical University, Xi’an, China; Politecnico di Milano, Italy

## Abstract

**Background:**

Artifical nerve scaffold can be used as a promising alternative to autologous nerve grafts to enhance the repair of peripheral nerve defects. However, current nerve scaffolds lack efficient microstructure and neurotrophic support.

**Methods:**

Microsphere–Scaffold composite was developed by incorporating chitosan microspheres loaded with nerve growth factor (NGF–CMSs) into collagen-chitosan scaffolds (CCH) with longitudinally oriented microchannels (NGF–CMSs/CCH). The morphological characterizations, *in vitro* release kinetics study, neurite outgrowth assay, and bioactivity assay were evaluated. After that, a 15-mm-long sciatic nerve gap in rats was bridged by the NGF–CMSs/CCH, CCH physically absorbed NGF (NGF/CCH), CCH or nerve autograft. 16 weeks after implantation, electrophysiology, fluoro-gold retrograde tracing, and nerve morphometry were performed.

**Results:**

The NGF–CMSs were evenly distributed throughout the longitudinally oriented microchannels of the scaffold. The NGF–CMSs/CCH was capable of sustained release of bioactive NGF within 28 days as compared with others *in vitro. In vivo* animal study demonstrated that the outcomes of NGF–CMSs/CCH were better than those of NGF/CCH or CCH.

**Conclusion:**

Our findings suggest that incorporation of NGF–CMSs into the CCH may be a promising tool in the repair of peripheral nerve defects.

## Introduction

Different types of artificial nerve scaffolds have been explored as alternatives to autografts to repair peripheral nerve defects [1]–[3]. Several empty nerve scaffolds have been approved for clinical use in peripheral nerve repair, such as Neurotube (polyglycolic acid) [4], NeuraGen (collagen type 1) [5], and Neurolac (poly(DL–lactide–ε–caprolactone)) [6]. However, the clinical and experimental outcomes of these empty nerve scaffolds remain unsatisfactory [7], [8]. The limited success of these scaffolds may be attributed to the lack of efficient microstructure and neurotrophic support for guiding the growth of regenerating nerves and promoting axonal regeneration.

Recently we have fabricated the collagen**-**chitosan scaffolds (CCH) [9]. The scaffold contains longitudinally orientated microchannels that are capable of guiding the linear growth of regenerating axons. However, the scaffold lacks neurotrophic support, which is another important factor in promoting nerve regeneration. Therefore, we speculate that incorporation of neurotrophic factors into the CCH hold great potential for promoting nerve regeneration.

Among various types of neurotrophic support, nerve growth factor (NGF), as an important member of neurotrophin family, not only promotes the survival and neurite outgrowth of sensory neurons, both *in vitro* and *in vivo*, but also enhances peripheral nerve regeneration, as shown in many previous studies [10], [11]. NGF can be physically absorbed into nerve scaffolds to be used for nerve regeneration. However, a general problem in using this method centers on the unfavorable initial burst release and limited bioactivity [12]. In our previous study, we developed chitosan microspheres loaded with NGF (NGF–CMSs) [13]. *In vitro* study has proven that NGF–CMSs were capable of sustained release of bioactive NGF. In the present study, we incorporated NGF–CMSs into the CCH to develop trophically and topographically functionalized microsphere–scaffold composite and investigated the feasibility of using the composite for bridging 15-mm-long sciatic nerve gap in rats.

## Methods

### Chitosan microspheres loaded with NGF (NGF–CMSs) preparation

NGF–CMSs were fabricated by a previously described, emulsion-ionic cross-linking method [13]. Briefly, chitosan solution (2%, w/v) was prepared by dissolving chitosan (Sigma, CA) in 10 ml of aqueous acetic acid solution (2%, v/v). 10 µg of NGF (R&D Systems Inc, Minneapolis, MN, USA) and 0.5 mg of bovine serum albumin (Sigma, CA) in phosphate-buffered saline (PBS, pH 7.4) were carefully added to the above solution, which was used as a water phase. 200 ml of liquid paraffin, containing surfactant span 80 (2%, v/v), was used as an oil phase (4°C). The water phase was then dropped slowly into the oil phase and stirred for 1 h at 4°C to form W/O emulsion. Thereafter, 20 ml of sodium tripolyphosphate solution (3% w/v; Sigma, CA) as the cross-linking agent was injected slowly into the W/O emulsion and stirred for 1 h. The NGF–CMSs were washed with petroleum petroleum ether and isopropyl alcohol, prior to lyophilization (Alpha 2–4, Chaist, Germany).

### Fabrication of the CCH, NGF/CCH, and NGF–CMSs/CCH

**Figure 1:**
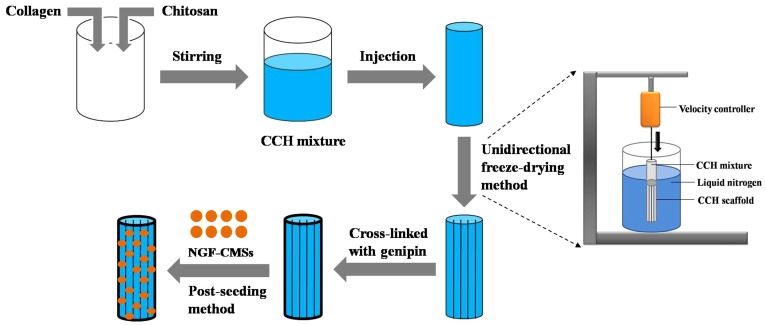
Schematic illustration of the basic steps in fabricating the NGF–CMSs/CCH.

The CCH was prepared using a unidirectional freezing method from our previous study (Figure 1) [9]. Briefly, type I collagen (2.5 wt%; Sigma, CA) and chitosan (0.5 wt%) were dissolved in a solution of acetic acid (0.05 M). The CCH mixture was stirred for 30 min and then injected into a cylindrical copper mold (50.0 mm in length and 2.0 mm in diameter). The mold was vertically placed into a nitrogen canister at a velocity of 4×10^−5^ m/s. After the CCH mixture was completely immersed in liquid nitrogen, it was lyophilized in a freeze–dryer at –80°C for 24 h. The dried scaffold was blocked with microtome blades into cylinders (15 mm in length and 2.0 mm in diameter). Subsequently, the scaffold was cross-linked by genipin solution (1 wt%; Challenge Bioproducts, Taichung, Taiwan) at 37°C for 48 h and then dried again by freeze–dryer.

The NGF–CMSs/CCH was prepared using a previously described post-seeding technique (Figure 1) [12]. Briefly, 70 mg of NGF–CMSs (NGF amount: 100 ng/scaffold) was suspended in 200 µl distilled water. 100 µl of the suspension was dropped carefully into the one side of the CCH. After that, another 100 µl of suspension was dropped carefully into the other side of the scaffold. This process was repeated three times until all of microsphere-containing suspension was incorporated into the composite. Finally, the microsphere-scaffold composite was lyophilized and stored at –20°C.

The NGF/CCH was fabricated using the same preparation method. Solutions of NGF (NGF amount: 100 ng/scaffold) in combination with bovine serum albumin (0.5 µg) in 200 µl distilled water were carefully dropped into the both sides of the CCH and then dried by freeze–dryer.

### Morphological characterization and microsphere size

The morphology of the NGF–CMSs, CCH and NGF–CMSs/CCH were evaluated by a scanning electron microscopy (S-3400N; HITACHI, Japan). The NGF–CMSs, CCH (longitudinal and transverse planes), and NGF–CMSs/CCH (longitudinal and transverse planes) were mounted on metal stubs using double-sided adhesive tape, coated with gold using a sputter coater, and then sequentially observed.

The particle mean size and size distribution of the microspheres were determined by a laser particle size analyzer (Mastersizer2000, UK). The dried microspheres were dispersed into deionized water and agitated on a shaker table at 300 rpm for more than 5 min. The obtained solution was used to determine mean size and size distribution.

### NGF release kinetics study

*In vitro* NGF release profiles from the NGF–CMSs/CCH were determined as follows [14]. In brief, the NGF–CMSs/CCH was dipped in 2.0 ml sterile PBS and kept in a shaking incubator (37°C, 40 rmp) for various time periods up to 28 days. At designated time point, the solution was centrifuged at 13,500 rmp. 200 µl of the release medium were withdrawn and frozen to –20°C until analysis by NGF enzyme-linked immunosorbent assay kit (ELISA; R&D Systems Inc, Minneapolis, USA), following the supplier's instructions. 200 µl of fresh medium were again added to the samples. The release experiment of NGF from the NGF/CCH was similar to that from the NGF–CMSs/CCH. The *in vitro* release experiments were performed in triplicate for each of the samples and the data were shown as mean ± SD.

### PC12 cell neurite outgrowth assay

To confirm the bioactivity of the released NGF from the NGF–CMSs/CCH, pheochromocytoma (PC12) cells were used as an assay system *in vitro* [15]. PC12 cells were cultured in T-75 flask at 1.0×10^5^ cells/ml in 10 ml culture medium consisting of RPMI1640, 15% horse serum, 2.5% fetal bovine serum and 1% Penicillin/Streptomycin. The cells were seeded at a density of 1.0×10^4^ cells/well in 1 ml culture medium on 24-well culture plate. At different times (1, 7, 14, and 28 days), a volume of 300 µl of the NGF supernatant released from NGF–CMSs/CCH was added to each well. Cultures were maintained at 37°C in 5% CO_2_ atmosphere for 72 h. Cells were viewed with an inverted phase contrast microscope (Nikon Instruments Inc., Melville, NY) and photographed. To quantify neurite extension, the lengths of the longest neurites of PC12 cells were measured using ImageJ software. Cells were counted when they met the criteria: neurites longer than the cell body. More than 100 cells were analyzed in each well. The neurite outgrowth assay of the NGF/CCH was similar to that of the NGF–CMSs/CCH. The control media was the released media without NGF.

### Cell viability (MTT) assay

Cell viability of PC12 cells in co-cultured various nerve scaffolds was determined by MTT (3-(4, 5-dimethylthiazol-2-yl)-2, 5-diphenyltetrazolium bromide) chromometry assay [16]. The cells were maintained in RPMI1640 and supplemented with 15% horse serum, 2.5% fetal bovine serum and 1% Penicillin/Streptomycin, at 37°C in a humid 5% CO_2_ incubator.

Each sample was first cut into several segments along the long axis and incubated in 2 ml of culturing medium at 37°C overnight. The medium was aspirated from the sample and 20 µl of cells suspension (1×10^5^ cells/well) were evenly seeded into each sample. After 2 hours of co-culture, 700 µl of culture medium was added into each well. The constructs were incubated at 37°C in a humid incubator. After 1, 7, 14 and 28 days of co-culture, the constructs were washed three times with PBS and then incubated at 37°C for 4 h in 20 µl of MTT and 700 µl of culture medium. After incubation, the media were removed and formazan crystals were added to water containing dimethyl sulfoxide, and the plates were shaken for 20 min. The plates were then read on a Microplate reader (Titertek, Helsinki, Finland) at 570 nm wavelength.

### Animals and surgical procedure

In this study a total of 80 adult male SD rats, weighing between 200 and 220 g, were used. All surgical procedures were conducted under a protocol reviewed and approved ethically by the Institutional Ethical Committee of the Fourth Military Medical University (approval ID: 2010010). In the first experimental group, for the study of fluoro-Gold (FG) retrograde tracing and electrophysiological assessment, 24 rats were randomly assigned to 4 subgroups: CCH, NGF/CCH, NGF–CMSs/CCH, and autograft groups. In the second experimental group, for the study of electrophysiological assessment and nerve morphometry, 56 rats were randomly assigned to 4 subgroups: CCH, NGF/CCH, NGF–CMSs/CCH and autograft groups. All rats were anesthetized by intraperitoneal injection of sodium pentobarbital solution (100 mg/ml). The left sciatic nerve was carefully exposed after making a skin incision and splitting the underlying muscles in the left lateral thigh. A segment of sciatic nerve was transected with microsurgery scissors, leaving a 15-mm-long gap after retraction of the nerve ends. The CCH, NGF/CCH, or NGF–CMSs/CCH group was then used to bridge the nerve stumps using 10/0 nylon sutures. In the autograft group, the nerve segment was removed and re-implanted in reverse direction across the nerve gap. After surgery, the rats were returned to their cages with free access to food and water.

### Electrophysiological assessment

In all animals of both experimental groups, electrophysiological tests were performed before tissue harvesting, 4, 8, 12, and 16 weeks after implantation. Briefly, under anesthesia with sodium pentobarbital, the left sciatic nerve was re-exposed by incision of previous surgical site. The nerve repair area was insulated from the surrounding muscle with a rubber dam. A bipolar stimulating electrode was placed under the sciatic nerve at a location 10 mm proximal to the graft site. A recording electrode was placed in the gastrocnemius muscle. Then the compound muscle action potentials (CMAP) were recorded with a Power Lab 4SP distal data acquisition system (Keypoint 3.02 Denmark). The peak amplitude of CMAP and nerve conduction velocity (NCV) values was calculated [17].

### Fluoro-Gold (FG) retrograde tracing

In the first experimental group, all animals were anesthetized for FG retrograde labeling assessment [18]. In brief, 16 weeks after implantation, 2 µl of 4% FG solution (Biotium Inc, CO) was injected into the rat sciatic nerve trunk at a point 5 mm distal to the grafts, and then the incision was sutured. After being kept routinely for 7 days, the rats had intracardiac perfusion with 4% (w/v) paraformaldehyde in 0.1 M phosphate buffer. After the vertebral canal was opened, the L4, L5, and S1 together with the dorsal root ganglia (DRG), were excised, post-fixed in buffered 4% paraformaldehyde for 4 hours, and then cryoprotected in 30% sucrose overnight at 4°C, followed by sectioning on a cryostat. Transverse sections (thickness, 25 µm) of the spinal cords were prepared from 12 rats (n = 3 in each group). Longitudinal sections (thickness, 20 µm) of the spinal cords were prepared from 12 rats (n = 3 in each group). Longitudinal sections (thickness, 16 µm) of DRG were prepared from all rats. All the sections were mounted on glass slides, viewed and photographed under a fluorescent microscope (Olympus BX-60, Japan). The total numbers of FG-labeled motoneurons in spinal cord and sensory neurons in DRG in all sections were counted.

### Nerve morphometry

After 16 weeks of implantation, in all animals of the second experimental group, the graft was rapidly harvested and fixed in a cold buffered 3% glutaraldehyde solution. The graft was then post-fixed in 1% osmium tetroxide in 0.1 M sodium cacodylate buffer (pH 7.3) for 1 hour at room temperature, dehydrated in ethanol, and embedded in resin. Transverse semi-thin (thickness 1.0µm) and ultra-thin sections (thickness 50.0 nm) were prepared from the distal portion of the graft. Semi-thin sections were stained with toluidine blue and studied under a light microscope (AH3, Olympus, Japan). Ultra-thin sections were stained with uranyl acetate and lead citrate, followed by observation under a transmission electron microscope (H-600, HITACHI, Japan). For quantitative analysis, five semi-thin sections and five ultra-thin sections from the distal portion of the graft were randomly collected and analyzed with an image analysis system (Analysis LS Professional, Olympus Soft Imaging Solutions) [19]. Five visual fields were selected for each section (one field at the center of the cross section and the other four to the top, bottom, right, and left of the central field). In each group, axonal regeneration was estimated by: (1) the total number of myelinated axons per nerve transverse section (M_tot_); (2) the total area of regenerated nerves (A_tot_); and (3) the diameter of myelinated axons. The degree of myelination was estimated by the axon-to fiber diameter ratio (G-ratio) observed.

### Statistical Analysis

All data were expressed as means ± standard deviation. One-way analysis of variance (ANOVA) followed by Tukey's post hoc test was used to determine the statistical differences between experimental groups, using the computer software PRISM (GraphPad Software). Values of *p*<0.05 were considered statistically significant.

## Results

### Morphology of the NGF–CMSs, CCH and NGF–CMSs/CCH

**Figure 2:**
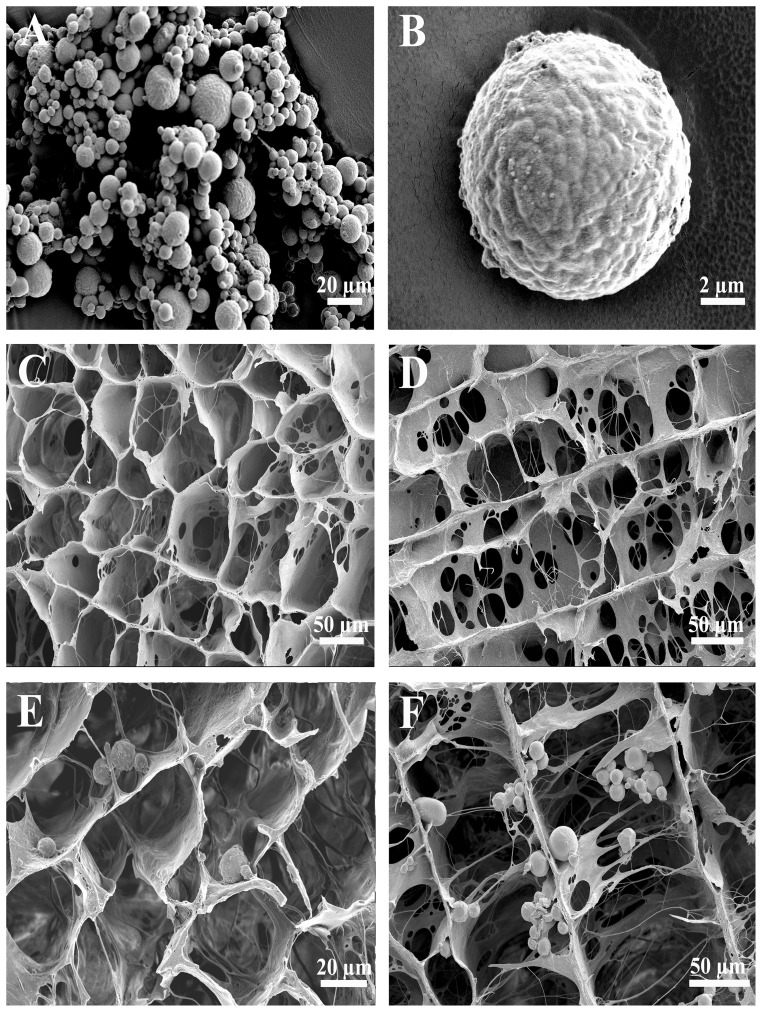
Microstructure appearance of the NGF–CMSs, CCH, and NGF–CMSs/CCH. Scanning electron microscopy images of the NGF–CMSs (A and B) show relative rough surface and spherical shape without hollows or deformations. Scanning electron microscopy images of the CCH scaffold in a transverse section (C) show that the microchannels were arranged in a honeycomb-like pattern. The longitudinal section of the CCH (D) shows the longitudinally oriented microchannels. Scanning electron microscopy images of the NGF–CMSs/CCH in a transverse section (E) show slightly different morphologies with pores, which were less regular and smaller than that of the CCH scaffold. The longitudinal section of the NGF–CMSs/CCH shows that the NGF–CMSs were evenly distributed throughout the longitudinally oriented microchannels which did not influence the microstructures of the scaffolds.

The NGF–CMSs showed relative rough surface and spherical shape without hollows or deformations (Figure 2A and B). The size distribution of NGF–CMSs varied from 2 to 50 µm based on the results of particle size analysis (data not shown). The mean size of NGF–CMSs was 18.8 µm which was suitable for incorporation of NGF–CMSs into the CCH. The transverse section of the CCH showed that microchannels were arranged in a honeycomb-like pattern (Figure 2C), and they had a mean cross-sectional diameter of 98.32±7.4 µm. The longitudinal section of the CCH showed that the scaffolds had longitudinally oriented microchannels (Figure 2D). The transverse section of the NGF–CMSs/CCH showed that microchannels were less regular and smaller than that of the CCH (Figure 2E), and they had a mean cross-sectional diameter of 90.58±2.8 µm. The longitudinal section of the NGF–CMSs/CCH showed that the NGF–CMSs were distributed throughout the microchannels in the CCH, which did not influence the microstructures of the scaffolds (Figure 2F).

### In vitro release study

**Figure 3:**
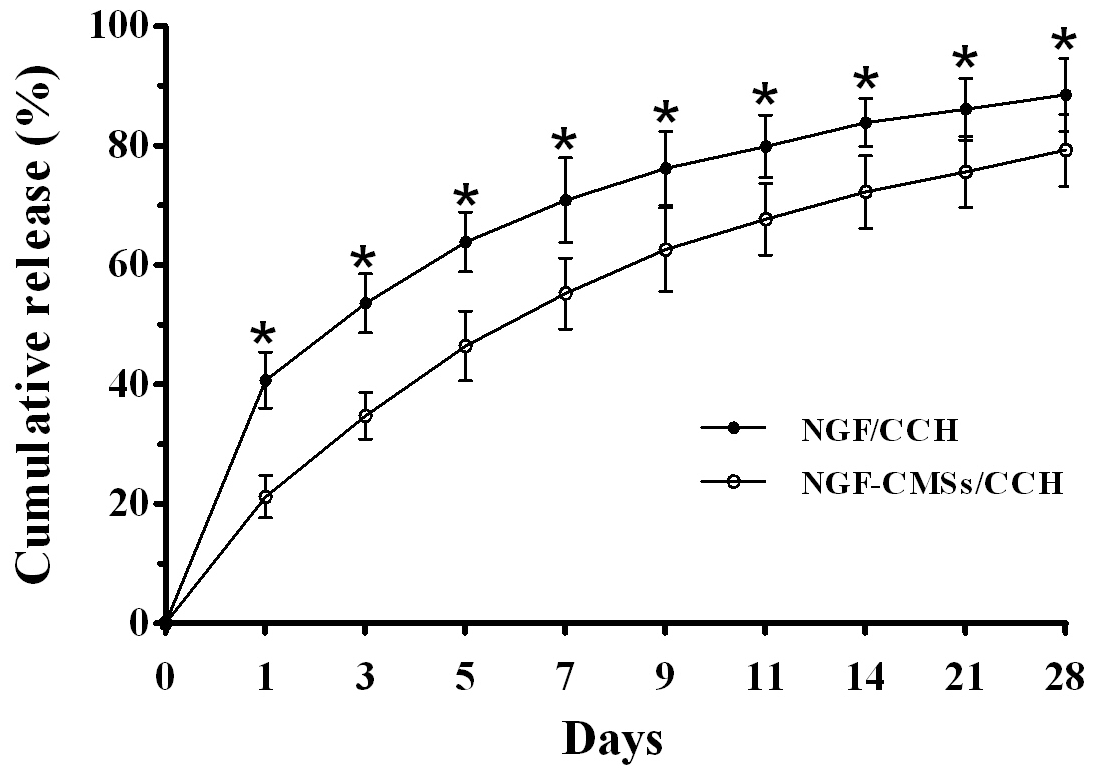
*In vitro* release kinetics of NGF from the NGF/CCH and NGF–CMSs/CCH over 28 days. All data represent mean ± SD (n = 3). *p<0.05 between NGF/CCH and NGF–CMSs/CCH.

To investigate the effect of NGF–CMSs incorporated into the CCH on NGF release, *in vitro* release kinetics of NGF from the NGF/CCH (NGF amount: 100 ng) and NGF–CMSs/CCH (NGF amount: 100 ng) was determined by ELISA (Figure 3). NGF release from the NGF/CCH and NGF–CMSs/CCH was extended over 28 days. However, the release rate of NGF from the NGF–CMSs/CCH was significantly lower than that from the NGF/CCH during 28 days (*p*<0.05). The main parameter that determined the NGF release was NGF–CMSs incorporated into the CCH, which lowered significantly the burst release during the initial 3 days and total amount of NGF released.

### Neurite outgrowth assay

**Figure 4:**
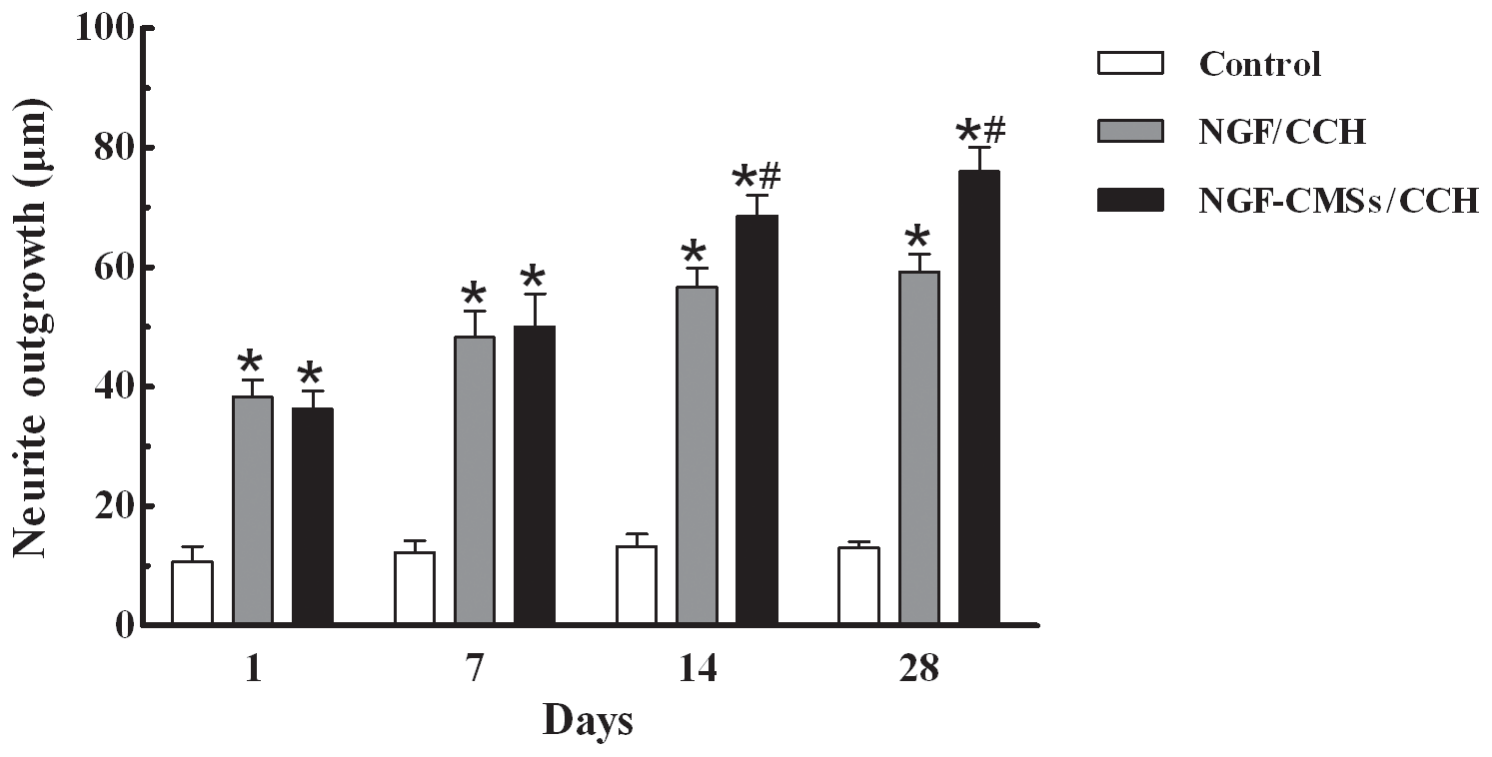
Neurite outgrowth assay. All data represent mean ± SD. *p<0.05 between control and others (NGF/CCH and NGF–CMSs/CCH). ^#^ *p*<0.05 between NGF/CCH and NGF–CMSs/CCH.

Neurite outgrowth assay was measured to evaluate the bioactivity of NGF released from the NGF/CCH and NGF–CMSs/CCH groups for 1, 7, 14 and 28 days (Figure 4). After addition of the release medium collecting 1 day and 7days, The length of neurite outgrowth in the NGF/CCH and NGF–CMSs/CCH groups were significantly longer than that in the control group (*p*<0.05), whereas no statistical difference was observed between NGF/CCH and NGF–CMSs/CCH groups (*p*>0.05). After addition of the release medium collecting 14 days and 28 days, the length of neurite outgrowth in the NGF–CMSs/CCH group was significantly longer than that in the NGF/CCH group (*p*<0.05).

### In vitro bioactivity assay

**Figure 5:**
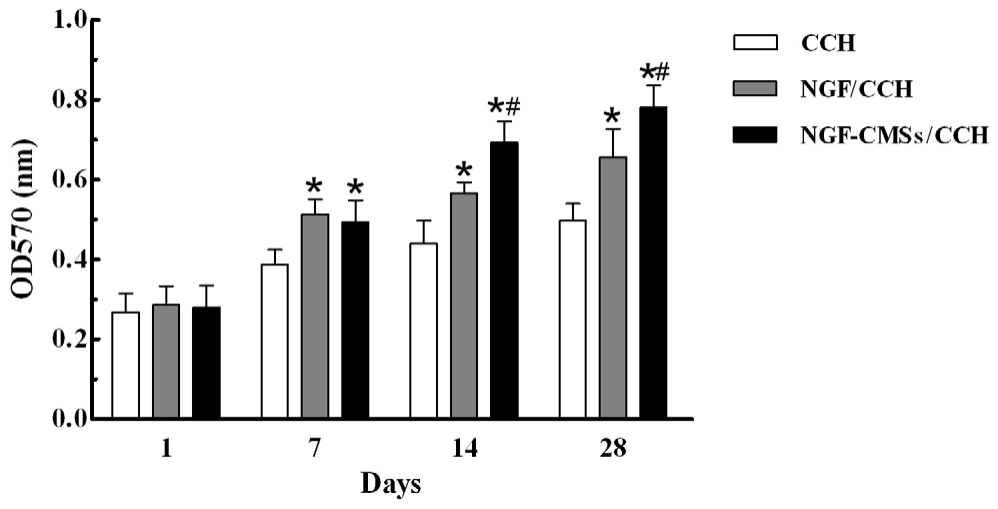
The MTT assay results. The PC12 cells which were co-cultured for 1, 7, 14, and 28 days in the CCH group (without NGF), NGF/CCH group (NGF amount: 100 ng), and NGF–CMSs/CCH group (microspheres amount: 70 mg, which contain 100 ng NGF). All data represent mean ± SD. *p<0.05 between CCH and others (NGF/CCH and NGF–CMSs/CCH). ^#^ *p*<0.05 between NGF/CCH and NGF–CMSs/CCH.

MTT chromometry assay was performed to determine the viability of PC12 cells co-cultured with the CCH, NGF/CCH, and NGF–CMSs/CCH groups for 1, 7, 14 and 28 days (Figure 5). After 1 day of co-culture, the three groups showed no significant difference from each other in terms of cell viability (*p*>0.05). However, after 7 days of co-culture, the cell viability in the NGF/CCH and NGF–CMSs/CCH groups were higher than that in the CCH group (*p*<0.05), whereas no statistical difference was observed between NGF/CCH and NGF–CMSs/CCH groups (*p*>0.05). After 14 days and 28 days of co-culture, the cell viability in the NGF–CMSs/CCH group was significantly higher than that in the NGF/CCH group (*p*<0.05).

### Electrophysiological assays

**Figure 6:**
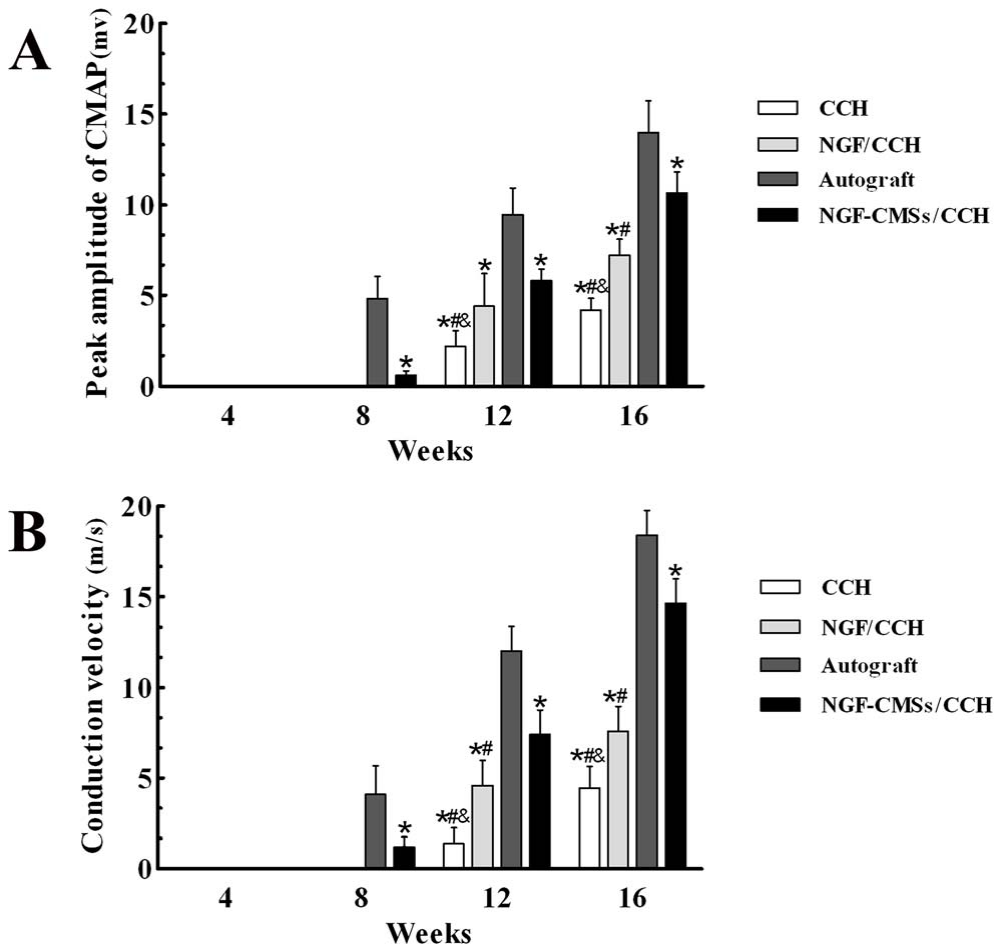
Electrophysiological assessments. All data represent mean ± SD. *p<0.05 between autograft and others (CCH, NGF/CCH, and NGF–CMSs/CCH). ^#^ *p*<0.05 between NGF–CMSs/CCH and others (CCH and NGF/CCH). ^&^ *p*<0.05 between NGF/CCH and CCH.

As shown in Figure 6A and B, the peak amplitude of CMAP and NCV values were detected 8 weeks after autograft and NGF–CMSs/CCH repair. At 8 weeks, the peak amplitude and NCV values of the autograft group were significantly higher than that of the NGF–CMSs/CCH group (*p*<0.05). At 12 weeks, the peak amplitude and NCV values of the autograft group were significantly higher than those of the other groups (*p*<0.05). The peak amplitude of the NGF–CMSs/CCH and NGF/CCH groups were significantly higher than that of the CCH group (*p*<0.05). There was no statistically significant difference between the NGF–CMSs/CCH and NGF/CCH groups (*p*>0.05). The NCV value of the NGF–CMSs/CCH group was significantly higher than those of the NGF/CCH and CCH groups (*p*<0.05). The NCV value of the NGF/CCH group was significantly higher than that of the CCH group (*p*<0.05). At 16 weeks, the peak amplitude and NCV values of the autograft group were significantly higher than those of the other groups (*p <*0.05). The peak amplitude and NCV values of the NGF–CMSs/CCH group were significantly higher than those of the NGF/CCH and CCH groups (*p*<0.05). The peak amplitude and NCV values of the NGF/CCH group were significantly higher than that of the CCH group (*p*<0.05).

### Fluoro-Gold (FG) retrograde tracing

**Figure 7:**
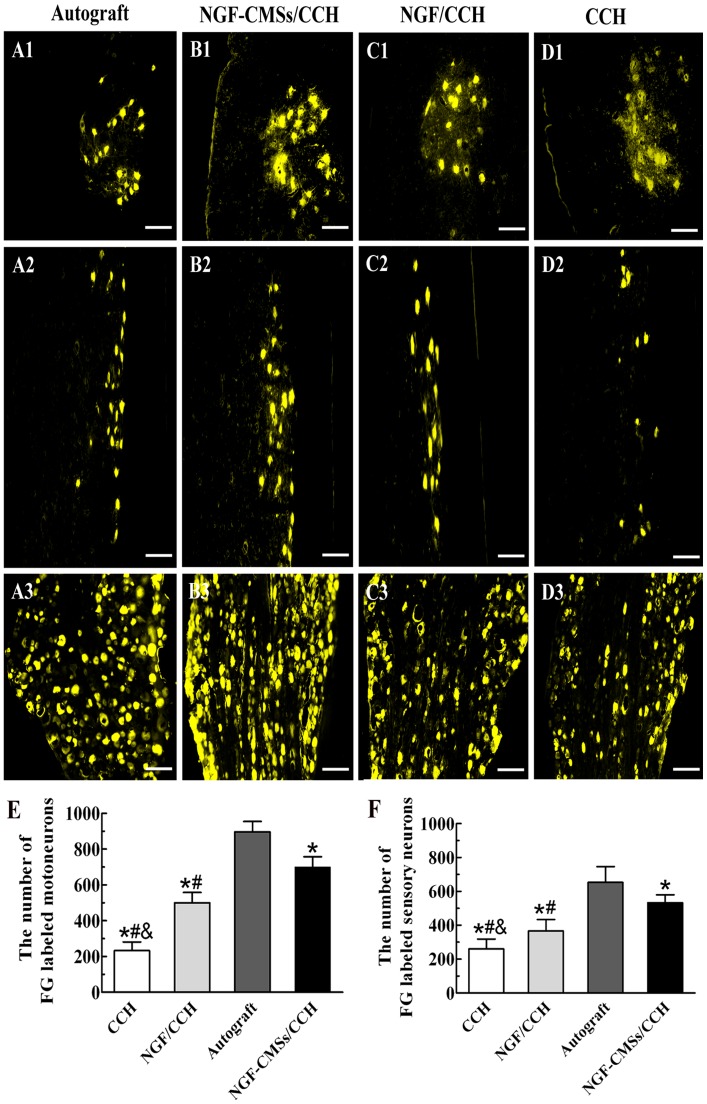
Fluoro-Gold (FG) retrograde tracing. Micrographs of FG-labeled motoneurons in spinal cord (transverse sections: A1, B1, C1, and D1; longitudinal sections: A2, B2, C2, and D2) and sensory neurons in dorsal root ganglions (transverse sections: A3, B3, C3, and D3) in the autograft group (A1–A3), NGF–CMSs/CCH group (B1–B3), NGF/CCH group (C1–C3), and CCH group (D1–D3) at 16 weeks after nerve-bridging operation. The total number of FG-labeled motoneurons and sensory neurons in each group were shown in (E) and (F). All data represent mean ± SD. *p<0.05 between autograft and others (CCH, NGF/CCH, and NGF–CMSs/CCH). ^#^ *p*<0.05 between NGF–CMSs/CCH and others (CCH and NGF/CCH). ^&^ *p*<0.05 between NGF/CCH and CCH. Scale bar = 100 µm.

FG-labeled motoneurons in spinal cord and sensory neurons in DRG were found in all the experimental groups 16 weeks after injection of FG (Figure 7A–D). As shown in Figure 7E and F,the number of FG-labeled motoneurons and sensory neurons in the autograft group was significantly higher than those of the other groups (*p*<0.05). The number of FG-labeled motoneurons and sensory neurons in the NGF–CMSs/CCH group was significantly higher than those of the NGF/CCH and CCH groups (*p*<0.05). The number of FG-labeled motoneurons and sensory neurons in the NGF/CCH group was significantly higher than that of the CCH group (*p*<0.05).

### Nerve morphometry

**Figure 8:**
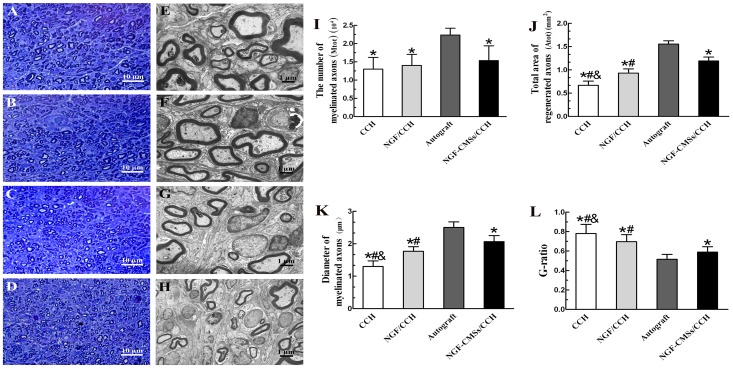
Morphometric analyses of regenerated nerves. Toluidine blue staining of regenerated axons at the distal portion (A–D) and transmission electron micrographs of regenerated axons at the distal portion (E–H) in the autograft group (A and E), NGF–CMSs/CCH composite group (B and F), NGF/CCH group (C and G), and CCH group (D and H). Morphometric evaluations of regenerated nerves at the distal portion were performed at 16 weeks after nerve-bridging operation. (I) The number of myelinated axons (M_tot_), (J) the total area of regenerated axons (A_tot_), (K) the diameter of myelinated axons, and (L) the G-ratio were measured from the distal section of the regenerated nerves. All data represent mean ± SD. *p<0.05 between autograft and others (CCH, NGF/CCH, and NGF–CMSs/CCH). ^#^ *p*<0.05 between NGF–CMSs/CCH and others (CCH and NGF/CCH). ^&^ *p*<0.05 between NGF/CCH and CCH. Scale bar = 10 µm for A, B, C, and D; scale bar = 1µm for E, F, G, and H.

Successful regeneration (defined for the presence of myelinated axons at the distal portion of nerve scaffolds) was observed in all the second experimental groups 16 weeks after implantation (Figure 8A–H). M_tot_ was not significantly different among nerve scaffold groups (*p*>0.05; Figure 8I), but was significantly higher after autograft repair (*p*<0.05). A_tot_ and the diameter of myelinated axons of the autograft group were significantly higher than those of the other groups (*p*<0.05; Figure 8J and K). A_tot_ and the diameter of myelinated axons of the NGF–CMSs/CCH group were significantly higher than those of the NGF/CCH and CCH groups (*p*<0.05; Figure 8J and K). A_tot_ and the diameter of myelinated axons of the NGF/CCH group were significantly higher than that of the CCH group (*p*<0.05; Figure 8J and K). In addition, G-ratio of the autograft group was significantly better than those of the other groups (*p*<0.05; Figure 8L). G-ratio of the NGF–CMSs/CCH group was significantly better than those of the NGF/CCH and CCH groups (*p*<0.05; Figure 8L). The G-ratio of the NGF/CCH was significantly better that of the CCH group (*p*<0.05; Figure 8L).

## Discussion

The purpose of the present study was to develop trophically and topographically functionalized microspheres–scaffold composite by incorporating NGF–CMSs into the CCH for bridging 15-mm-long sciatic nerve gap in rats. As with most biological processes, axonal growth depend on neurotrophic support and topography guide. In previous work, we have demonstrated that NGF–CMSs were capable of releasing bioactive NGF on axonal outgrowth *in vitro* [13]. Therefore, we assume that incorporating NGF–CMSs into the CCH (1) was the minimizing of the burst release of bioactive NGF from the CCH, as excessive initial NGF-doses can hamper the early axonal regeneration [20]; (2) will be more beneficial for axonal regeneration than the NGF/CCH or CCH.

The NGF–CMSs/CCH released NGF at a controlled rate over 28 days, with NGF remaining biologically active. Interestingly, the NGF–CMSs/CCH did not show an initial burst release, which is commonly seen with many other polymer based drug delivery systems. It may be due to NGF–CMSs degradation followed by the CCH degradation. Similar results have been reported by incorporating of BMP-2-derived synthetic containing chitosan microspheres into porous poly (lactic acid) scaffolds [14]. For NGF a very slow release should be biologically advantageous, as high amounts of NGF cause extensive branching of regenerating axons resulting in inappropriate target reinnervation and dysfunctional connections [21]. In addition, high amounts of NGF release delays early phase of axonal regeneration by delaying the neuronal perception of reduced levels of endogenous NGF caused by denervation [20]. Unfortunately, there are no studies available that are provide information of the optimal daily NGF dose over extended periods of time for peripheral nerve regeneration. Therefore, we did not intend to deliver an optimal concentration of NGF in the present study. Instead, we prepared the NGF–CMSs which could be incorporated into the CCH scaffold on a reproducible basis. Upon certifying the efficacy of this nerve guide model it will be possible to vary the weight of microspheres incorporated into the nerve scaffold for establishing an ideal dose of growth factor or adjusting microsphere formulations for improved loading capacity and encapsulation efficiency.

Regarding the bioactivity of growth factors, it is generally known that the *in vitro* half-life of many growth factors is very short. This half-life is reduced even further once growth factors are intravenously administered for *in vivo* experiments. It is thus necessary to protect growth factors that will be used *in vivo* from thermal denaturation, enzymatic degradation, and inactivation at acidic pH. Such protection has been shown to occur through chitosan microspheres embedding [22], [23]. In this study, we showed that the *in vitro* bioactivity of NGF released from the NGF–CMSs/CCH was maintained, as demonstrated by the neurite outgrowth and viability of PC12 cells. It may be attributed partly to the fact that microsphere incorporation can protect bioactive NGF from denaturing and partly to bioactive NGF and biodegradation products of chitosan, which may have a synergistic effect on maintaining cell viability [24].

The efficacy of NGF–CMSs/CCH in improving peripheral nerve regeneration was evaluated *in vivo* by functional and histological methods. The peak amplitude of CMAP and NCV values has been widely used for evaluating the conduction function of peripheral nerve. In this study, we found that the autograft group showed the highest peak amplitude of CMAP and NCV values. This is consistent with its highest number of regenerated axons. The NGF–CMSs/CCH group showed higher axonal diameter and myelination thickness than the other nerve scaffold group. More immature fibers were seen in the NGF/CCH and CCH groups with smaller fiber diameter which is related to the inferior peak amplitude of CMAP and NCV values. In addition, the number of FG-labelled motoneurons and sensory neurons in the NGF–CMSs/CCH group was significantly higher than those of the NGF/CCH and CCH groups. These findings thus suggest that more axons may successfully regenerate through the NGF–CMSs/CCH into the distal stumps and restoration of axonal transport function of regenerated nerve fibers. Our study corroborates previous findings in the literature on the effect of bioactive NGF on peripheral nerve regeneration [25]–[27]. Xu et al. evaluated the effects of NGF delivery from nerve guide conduits across a 10 mm rat sciatic nerve defect. Similar to our results, after 3 months implantation, they found that NGF delivery increased fiber diameter and fiber population at the end of regenerated nerve conduit, as compared with either saline alone or NGF protein without microencapsulation. In addition, Lee et al. evaluated the axonal regeneration across a 13-mm-long gap in the rat sciatic nerve in the presence of NGF provided the heparin-containing delivery system. They found that the total number of nerve fibers at the mid-conduit level showed no statistical difference for NGF from the isograft.

The beneficial effect of NGF–CMSs on nerve regeneration can be explained by several possible mechanisms. Firstly, NGF–CMSs incorporated into the CCH as a growth factor reservoir may play an important role in stabilizing the bioactivity of NGF. In contrast, NGF absorbed the CCH may lose bioactivity rapidly [28]. Secondly, the NGF–CMSs/CCH is able to release bioactive NGF in a controlled manner for at least 28 days *in vitro*. Thirdly, a continued supply of NGF from NGF–CMSs could enhance regeneration in motor nerves, as well as sensory nerves [29]. This might be explained by the fact that bioactive NGF would increase expression of high-affinity TrkA NGF receptors [30]. A potential outcome of increased expression of NGF receptors would be a further increase in the retrograde transport of NGF to the neurons and subsequent neuronal survival and regeneration of transected sciatic nerve in rodents [25]. In brief, the experimental data increase the feasibility of translation to clinical trials using NGF–CMSs as neurotrophic support for peripheral nerve injury.
